# Evaluation of cerebrospinal fluid levels for ALOX5, S100B, DEFA1, and GFAP in infectious meningitis

**DOI:** 10.1097/MD.0000000000036463

**Published:** 2023-12-15

**Authors:** Ali İrfan Baran, Zübeyir Huyut, Mehmet Reşit Öncü, Halil İbrahim Akbay, Şükrü Akmeşe, Hasan Karsen, Hamit Hakan Alp, Zekiye Hakseven Karaduman, Tayyar Tarcan

**Affiliations:** a Department of Infectious Diseases and Clinical Microbiology, Faculty of Medicine, Van Yuzuncu Yil University, Van, Turkey; b Department of Biochemistry, Faculty of Medicine, Van Yuzuncu Yil University, Van, Turkey; c Department of Emergency Medicine, Faculty of Medicine, Van Yuzuncu Yil University, Van, Turkey; d Departmant of Pharmacy Services Program, Vocational School of Health, Harran University, Sanliurfa, Turkey; e Department of Infectious Diseases and Clinical Microbiology, Faculty of Medicine, Harran University, Sanliurfa, Turkey; f Bitlis State Hospital, Bitlis, Turkey.

**Keywords:** aseptic, bacterial, cerebrospinal fluid, meningitis, tuberculosis

## Abstract

**Background:**

The aim of this study was to determine how the levels of peptide and protein-based biomarkers in cerebrospinal fluid change in bacterial, tuberculous, and aseptic meningitis, and to determine the success of these agents in distinguishing between different types of infectious meningitis.

**Methods:**

The levels of arachidonate-5-lipoxygenase, S100 calcium-binding protein B, defensin-α 1, and glial fibrillary acidic protein in cerebrospinal fluid samples from 20 tuberculosis, 40 bacterial, 25 aseptic meningitis patients, and 55 control groups were measured and compared using an enzyme-linked immunosorbent assay.

**Results:**

The mean age of the patients was 37.9 ± 14.4 years. The parameter that contributed the most to the differential diagnosis of the infectious meningitis groups was S100 calcium-binding protein B. The S100 calcium-binding protein B levels were significantly higher in the tuberculous meningitis group than in the other groups, and arachidonate-5-lipoxygenase levels were significantly higher in the tuberculous meningitis and bacterial meningitis groups (*P* < .05).

**Conclusion:**

This study showed that cerebrospinal fluid arachidonate-5-lipoxygenase, and S100 calcium-binding protein B levels may differ in bacterial, aseptic, and tuberculous meningitis, and the results obtained may be quite effective as important potential biomarkers in the differential diagnosis of different types of meningitis.

## 1. Introduction

Infectious meningitis is a central nervous system (CNS) infection that kills approximately half a million people worldwide and causes a variety of sequelae in millions of people.^[1]^ Infectious meningitis can be caused by bacterial, viral, fungal, and parasitic agents, which vary as a function of geographical region and age group.^[2,3]^ Acute bacterial meningitis is life-threatening and causes approximately 1.2 million cases and more than 100,000 deaths annually.^[4,5]^ Despite vaccination strategies, antibiotic therapy, and modern health care, mortality and morbidity rates remain high in both developing and developed countries. Cerebrospinal fluid (CSF) analysis including Gram stain, culture, and other markers, can help in diagnosis, but types of meningitis are not easily diagnosed. Aseptic (viral) meningitis (AM) usually resolves within a week or two without any treatment. However, bacterial meningitis involves long hospital stays and high costs.^[5–8]^ Tuberculous meningitis (TBM) has the worst prognosis and highest mortality rate. Approximately 50% of the TBM survivors develop neurological disabilities.^[7,8]^ Early recognition of infectious meningitis and appropriate treatment can prevent the disease from progressing, and reduce its severity, and mortality. Therefore, there is a need for biomarkers to clarify and accelerate differential diagnosis.^[6–8]^ Unnecessary and empirical use of antibiotics without differential diagnosis can lead to bacterial resistance increase and the risk of permanent sequelae development. Despite the use of various biomarkers in the CSF and serum, it is not always possible to distinguish between viral, bacterial, and tuberculous meningitis.^[9–11]^

Arachidonate-5-lipoxygenase (ALOX5) is an enzyme involved in the leukotriene B4 and lipoxin metabolic pathways. It plays a role in the pathogenesis of TBM as well as in the pathophysiology of asthma and certain cancers.^[12–16]^

S100 calcium-binding protein B (S100B) is a calcium-binding protein physiologically produced by CNS astrocytes. Some studies have shown that CSF and serum levels of S100B increase in various neurological disorders. S100B also plays a role in the pathogenesis of sepsis-associated encephalopathy and bacterial meningitis (BM) in children. However, there are limited data on how CSF S100B changes in meningitis and other brain infections.^[17–19]^

Defensins, also known as human neutrophil peptides, are small molecular weight cationic peptides with broad antibacterial, antiviral, and antifungal activities.^[20]^ Defensin-alpha 1-3 (DEFA1-3) is mainly found in neutrophils and its main target is phagolysosomes in granulocytes and monocytes, although it is also secreted extracellularly and circulates.^[21,22]^

Glial fibrillary acidic protein (GFAP) is an interim filament protein that is not always found in blood circulation and is released by astrocytes following injury to the glia. GFAP is a highly specific brain protein. Serum levels of GFAP, which are not always found in the bloodstream of healthy individuals, are quite low. GFAP is released by astrocytes following glial damage, and high serum levels may be a useful prognostic indicator of brain tissue damage, facilitating early detection of various neurological diseases.^[23–25]^

This information provides important clues that Defensin-alpha1 (DEFA1), ALOX5, S100B, and GFAP may also be associated with meningitis. Here, the levels of ALOX5, S100B, DEFA1, and GFAP in the CSF of patients with meningitis caused by different infectious agents were compared to those of healthy controls. Our aim was to investigate whether these molecules could be used as biomarkers to distinguish between different types of infectious meningitis and to aid in the early diagnosis of the disease.

## 2. Material and methods

### 2.1. Design of the study

This study was prospectively designed and conducted over a 24-month period in a university hospital. Patients who presented to the Infectious Diseases Clinic with complaints of fever, headache, neck stiffness, vomiting, and altered consciousness and were followed up with a diagnosis of meningitis were included in the study. The diagnosis of meningitis was made by evaluating the patient’s clinical condition, physical examination findings, CSF biochemistry obtained by lumbar puncture (LP), Gram stain and culture results. The first CSF samples collected before the start of treatment were included in the study. CSF examinations included CSF biochemistry, CSF microscopy/cell count, Gram stain, acid-fast stain, CSF Wright test, CSF culture, tuberculin skin test, CSF adenosine deaminase test, interferon-gamma release assay, tuberculosis PCR, and tuberculosis culture.

Fundus examination was performed in all patients, and contraindications to LP were excluded by brain computerized tomography imaging. Forty patients diagnosed with a BM, 25 with AM, and 20 with a TBM were included in the study. As a control group, 55 people with nonspecific complaints and normal CSF and other tests who had undergone LP with meningitis or other preliminary diagnoses were included in the study after obtaining their consent. The inclusion criteria for the study were patients older than 18 years of age and diagnosed with isolated infectious meningitis. The exclusion criteria included patients with any chronic disease, chronic medication use (especially antibiotic use) and those who refused LP. Patients were enrolled in the study after obtaining informed consent. The lumbar region was sterilized according to the LP procedure, and spinal needle intervention was performed from the L3-4 vertebral space. CSF samples were centrifuged at 2000 rpm for 10 minutes, transferred to Eppendorf tubes, and stored at −80°C. After sufficient samples were collected, they were thawed at room temperature for analysis and their levels were analyzed by enzyme-linked immunosorbent assay (ELISA). Ethics committee approval was obtained from the Ethics Committee of Harran University Faculty of Medical on 05.10.2017, session number 10, decision number 06.

### 2.2. ELISA analysis

ALOX5, S100B, DEFA1, and GFAP concentration estimation kits were used according to the manufacturer’s instructions (MyBioSource, San Diego, CA) and a standard ELISA protocol.

### 2.3. Statistical analysis

Statistical analysis of the data was performed using SSPS 22.0 (version 22; SPSS Inc., Chicago, Il, USA).0. Results are presented as the mean ± standard deviation for continuous variables. Normality analysis was performed using the Shapiro–Wilk test and histogram graph. Differences between groups were evaluated with one-way analysis of variance, and pairwise group comparisons were checked with Tukey’s or Games-Howell test using Levene’s test for homogeneity of variance. Statistical significance was set at *P* < .05.

For multivariate statistical analysis, data from the patient and control groups were uploaded to MetaboAnalyst 5.0 server (https://www.metaboanalyst.ca/). Principal Component Analysis (PCA) was used to visualize the segregation and clustering of individuals into groups. Variable importance in projection (VIP) scores were calculated for candidate biomarkers (ALOX5, S100B, DEFA1, and GFAP) that contributed to differentiation between groups. We also generated heat maps of the groups for these potential biomarkers by hierarchical clustering using the Euclidean distance measure, *t* test mode, and Ward’s algorithm. The Biomarker Analysis module, based on MetaboAnalyst’s receiver operating characteristic (ROC) curve, was used to identify potential biomarkers that could be used to diagnose patients with meningitis infections caused by different infectious factors.

## 3. Results

The mean age of the BM group included in the study was 40.5 ± 14.3 years, the mean age of the AM group was 28.6 ± 8.8 years, the mean age of the TM group was 44.1 ± 15.5 years, and the mean age of the control group was 37.9 ± 12.6 years. Other demographic data are detailed in Table 1. CSF ALOX5, S100B, DEFA1, and GFAP levels were analyzed according to the results of post hoc analysis. We then separated of groups by PCA analysis, screening for differential data, and heat map analysis. Levels were found to be higher in the infectious meningitis groups than in the control group (*P* < .05).

**Table 1:** Demographic data of meningitis and control groups.

Descriptive analysis	Groups				*P*	Pairwise comparisons
	Control group (n = 55)	Bacterial meningitis (n = 40)	Aseptic meningitis (n = 25)	Tuberculosis meningitis (n = 20)		
Age	37.9 ± 12.64	40.50 ± 14.34	28.64 ± 8.84	44.10 ± 15.49	<.01*	AM-BM‡
Min.	18	18	18	18		AM-TBM‡
Max.	65	67	48	69		AM-Control group‡
Gender						
Male	33 (60)	25 (62.5)	14 (56)	12 (60)	.965†	
Female	22 (40)	15 (37.5)	11 (44)	8 (40)		

AM = aseptic meningitis, BM **=** bacterial meningitis, Max. **=** maximum, Min. **=** minimum, TBM = tuberculous meningitis.

* Comparison among four groups by Kruskal–Wallis test.

† Comparison among four groups by Chi-squared test.

‡ Pairwise comparison with Mann–Whitney *U* test (*P* < .05).

### 3.1. CSF ALOX5, S100B, DEFA1 and GFAP post hoc analysis results

Although CSF ALOX5 levels were higher in the TBM and BM groups than in the control group (*P* < .05), the same parameter value in the AM group was similar to that in the control group. There was no significant difference between them (*P* > .05). Simultaneously, S100B levels were higher in the TBM and BM groups than in the control and aseptic meningitis groups (*P* < .05). DEFA1 and GFAP levels were significantly higher in the TBM, BM, and AM groups than in the control group. Means and standard deviations of ALOX5, S100B, DEFA1, and GFAP levels and post hoc analysis groups are shown in Table 2.

**Table 2:** Comparison of CSF levels of ALOX5, S100B, DEFA1, GFAP in the groups with infectious meningitis and in the healthy control groups.

	Group (Mean ± SD)				*P*	Pairwise comparisons
	Control group (n: 55)	Tuberculous meningitis (n: 20)	Bacterial meningitis (n: 40)	Aseptic meningitis (n: 25)		
ALOX5	0.11 ± 0.06	0.19 ± 0.01	0.14 ± 0.06	0.11 ± 0.06	.001*	‡TBM-Control group; ‡BM-Control group; ‡TBM-BM; ‡TBM-AM; ‡BM-AM;
S100B	1.41 ± 0.59	3.68 ± 1.61	1.57 ± 0.67	1.36 ± 0.68	<.001†	§TBM-AM; §TBM-BM; §TBM-Control group
DEFA1	480.97 ± 136.64	1098.72 ± 633.45	1064.85 ± 576.72	950.81 ± 429.66	<.001†	§Control group-AM §Control group-BM §Control group-TBM
GFAP	4.02 ± 0.96	8.91 ± 3.69	7.87 ± 2.86	8.42 ± 2.18	<.001*	‡Control group-AM ‡Control group-BM ‡Control group-TBM

ALOX5 = Arachidonate-5-lipoxygenase, AM **=** Aseptic meningitis, BM **=** Bacterial meningitis, CSF **=** Cerebrospinal fluid, DEFA1 **=** Defensin alpha-1, GFAP **=** Glial fibrillary acidic protein, S100B **=** S100Calcium-binding protein B, TBM = Tuberculous meningitis.

* Comparison among four groups by Kruskal–Wallis test.

† Comparison among four groups by one-way analysis of variance.

‡ Pairwise comparison with Mann–Whitney *U* test (*P* < .05).

§ Pairwise comparison with Tukey test (*P* < .05).

### 3.2. Separation of groups with PCA analysis

**Figure 1.**
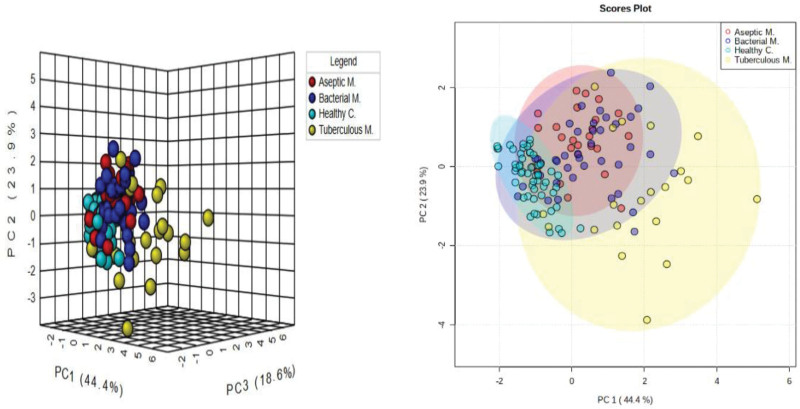
Two-dimensional (A) and three-dimensional (B) PCA score plots of the groups. Aseptic M.: Aseptik meningitis, Bacterial M.: Bacterial Menengitis, Tuberculous M.: Tuberculous Menengitis, Healthy C.: Healthy Control. PCA = Principal Component Analysis.

**Figure 2.**
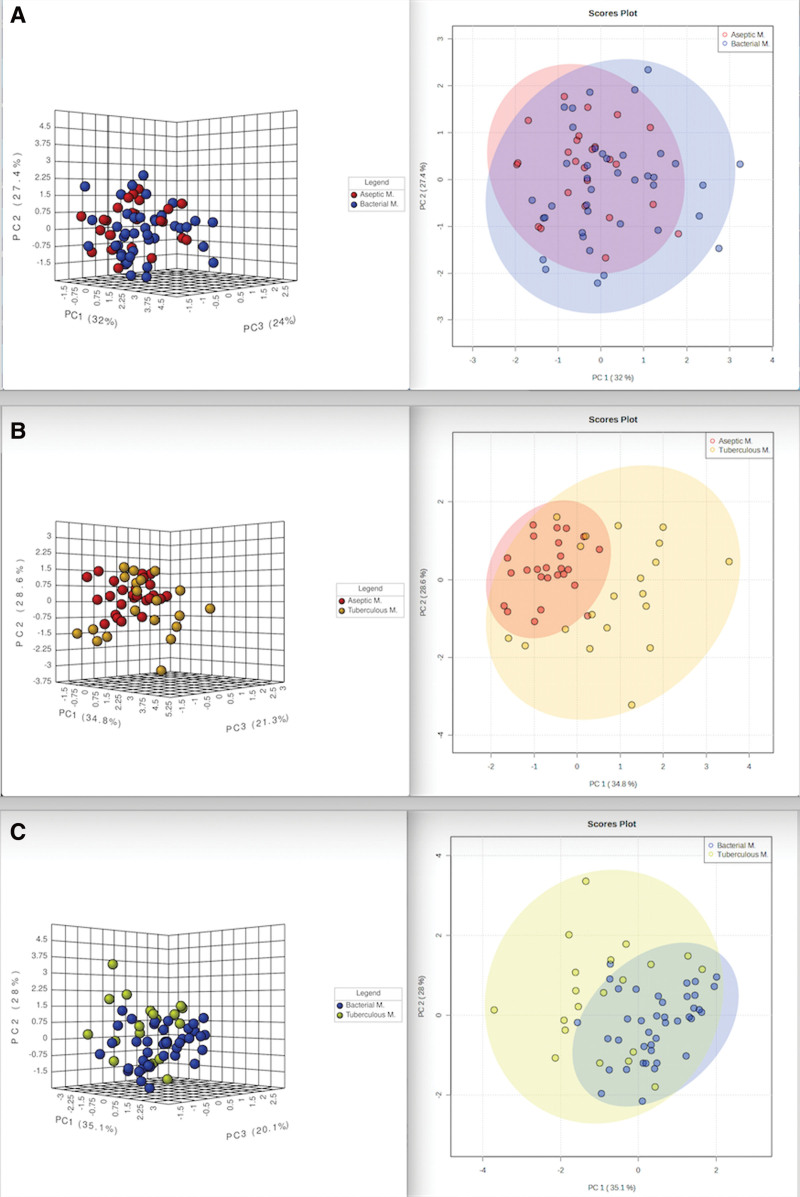
Two-dimensional and three-dimensional PCA score plots of the groups diagnosed with meningitis. (A) Aseptic meningitis and bacterial meningitis (B) Aseptic meningitis and tuberculous meningitis (C) Bacterial meningitis and tuberculous meningitis. Aseptic M.: Aseptik meningitis, Bacterial M.: Bacterial Menengitis, Tuberculous M.: Tuberculous Menengitis. PCA = Principal Component Analysis.

PCA was used to visualize the distribution of individuals in the different meningitis and control groups. The analysis results are presented in two and three dimensions, respectively. According to the PCA results, although there was partial separation between the groups, significant clustering was observed between the groups. DEFA1, GFAP, ALOX5, and S100B successfully distinguished patients with meningitis from controls. When comparing all meningitis groups (BM, AM, and TBM), a significant clustering was observed although there was no clear differentiation (Figs. 1 and 2).

### 3.3. Screening for differential data

**Figure 3.**
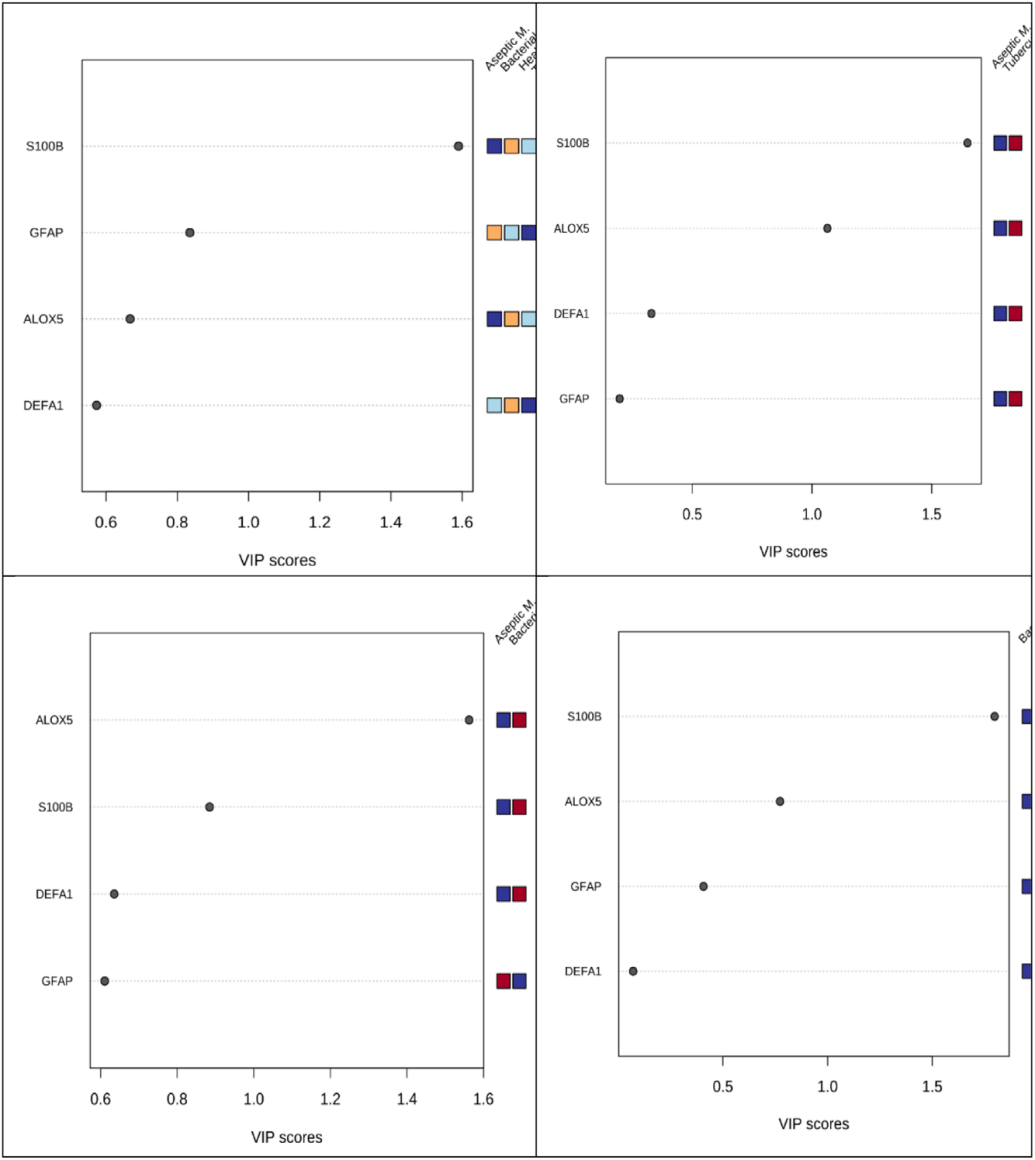
VIP score graph of all groups: Their ability to discriminate patients from healthy subjects in descending order of importance. Aseptic M.: Aseptik meningitis, Bacterial M.: Bacterial Menengitis, Tuberculous M.: Tuberculous Menengitis, Healthy C.: Healthy Control.

VIP plots were generated to determine the contribution of ALOX5, S100B, DEFA1, and GFAP to the decomposition of the groups (Fig. 3). A higher VIP score implied a greater contribution to the decomposition. The burgundy color indicates up-regulation and the blue color indicates down-regulation. S100B had the highest contribution to the differentiation of meningitis groups from the healthy control group. In addition, the degree of contribution to differentiating the types of meningitis was used to determine VIP score plots. When comparing the aseptic, bacterial, and tuberculous meningitis groups, ALOX5 and S100B showed the highest VIP scores.

### 3.4. Heatmap findings

**Figure 4.**
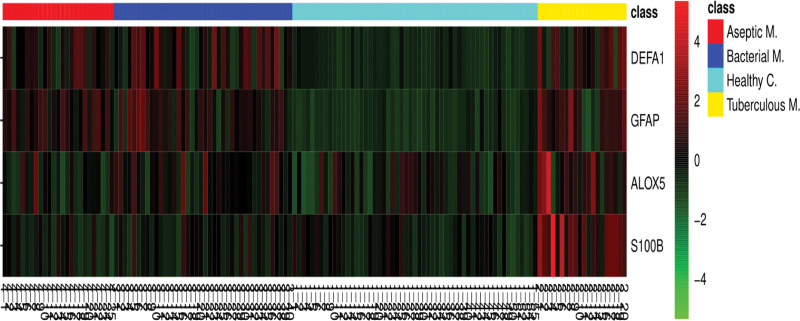
Heatmap of CSF ALOX5, S100B, DEFA1 and GFAP levels. ALOX5 = Arachidonate 5-lipoxygenase, CSF = Cerebrospinal fluid, DEFA1 = Defensin alpha-1, GFAP = Glial fibrillary acidic protein, S100B = S100 calcium-binding protein B. Aseptic M.: Aseptik meningitis, Bacterial M.: Bacterial Menengitis, Tuberculous M.: Tuberculous Menengitis, Healthy C.: Healthy Control.

A heat map was created to visualize the concentrations of DEFA1, GFAP, ALOX5, and S100B in the meningitis and healthy control groups (Fig. 4). Red bands indicate an increase in the relevant biomarker within the group, whereas green bands indicate a decrease. According to the heat map, ALOX5, S100B, DEFA1, and GFAP levels were partially increased in the meningitis group versus controls. The levels of ALOX5 and S100B were significantly higher in the TBM group than in other groups. The levels of DEFA1 and GFAP were significantly lower in the control group than in other groups.

### 3.5. Potential biomarkers for meningitis types

ROC curve analysis was performed to differentiate the variation in ALOX5, S100B, DEFA1, and GFAP between groups to distinguish meningitis patients from healthy individuals and from different types of meningitis. Comparison between groups: Results by cutoff, area under the curve (AUC), sensitivity, specificity, positive likelihood ratio, and negative likelihood ratio are shown in Table 3.

**Table 3:** Comparison between groups; cutoff, area under the curve (AUC), sensitivity, specificity, positive likelihood ratio (LR+), and negative likelihood ratio (LR−).

Comparisons	Biomarkers	Cutoff value	AUC	Sensitivity	Specificity	LR	LR−
Bacterial meningitis and aseptic meningitis	ALOX5	0.12	0.677	0.57	0.68	1.796	0.625
	S100B	1.56	0.586	0.50	0.68	1.562	0.735
	GFAP	8.95	0.554	0.65	0.44	1.160	0.795
	DEFA1	856	0.552	0.62	0.44	1.116	0.852
Aseptic meningitis and control group	ALOX5	0.12	0.513	0.41	0.64	1.161	0.909
	S100B	1.4	0.534	0.56	0.60	1.409	0.727
	GFAP	5.13	0.983	0.89	1.0	Infinity	0.109
	DEFA1	731	0.882	0.96	0.72	3.441	0.050
Bacterial meningitis and control group	ALOX5	0.118	0.630	0.58	0.70	1.939	0.597
	S100B	1.62	0.560	0.63	0.47	1.212	0.765
	GFAP	5.59	0.882	0.92	0.80	4.636	0.090
	DEFA1	732	0.809	0.96	0.72	3.504	0.050
Tuberculous meningitis and aseptic meningitis	ALOX5	0.151	0.772	0.70	0.88	5.833	0.340
	S100B	2.02	0.936	0.90	0.84	5.625	0.119
	GFAP	9.05	0.574	0.70	0.64	1.944	0.468
	DEFA1	1140	0.578	0.55	0.68	1.718	0.661
Tuberculous meningitis and control group	ALOX5	0.15	0.758	0.70	0.74	2.750	0.402
	S100B	2.02	0.933	0.90	0.9	8.250	0.112
	GFAP	7.07	0.839	0.80	1.0	Infinity	0.200
	DEFA1	593	0.771	0.75	0.80	3.750	0.312
Bacterial meningitis and tuberculous meningitis	ALOX5	0.149	0.670	0.70	0.72	2.545	0.413
	S100B	2.15	0.906	0.85	0.85	5.666	0.176
	GFAP	8.97	0.608	0.70	0.65	2.000	0.461
	DEFA1	1140	0.516	0.55	0.62	1.466	0.720

ALOX5 = arachidonate-5-lipoxygenase, AUC = area under the curve, DEFA1 = defensin alpha-1, GFAP = glial fibrillary acidic protein, LR− = negative likelihood ratio, LR+ = positive likelihood ratio, S100B = S100Calcium-binding protein B.

## 4. Discussion

Bacterial meningitis and tuberculous meningitis can have a more severe clinical course and can lead to brain damage, disorders of the nervous system, and even fatal outcomes. Therefore, accurate methods to identify meningitis using molecular markers at an early stage of infection are critical for effective treatment.^[15,26]^ ALOX5 activity is a rate-limiting enzyme responsible for the biosynthesis of leukotrienes, which are essential for inflammatory responses. These important mediators play a role in the development of many diseases.^[27]^ In related literature studies, Kataria et al performed proteomic analysis and two-dimensional electrophoresis on CSF from patients with and without TBM. They found that ALOX5 had high specificity for TBM with increased concentrations (*P* < .01).^[15]^ In our study, ALOX5 levels were higher in the TBM and BM groups than in the control group, and there was an important difference between them (*P* < .05). However, we could not find a sufficient number of studies that showed an association between infectious meningitis and ALOX5.

The level of S100B protein is associated with the severity of brain injury and the outcome after brain injury. It is also considered to be the C-reactive protein in the brain. The S100B protein is a biomarker known to indicate neuronal damage in vascular brain disease, stroke, and brain injury.^[18,19,26,28,29]^ Studies in children with bacterial meningitis showed an increase in the level of S100B in serum, and CSF and S100B could be used to diagnose of bacterial meningitis in children. CSF S100B is a marker of damage in purulent meningitis and has been implicated as useful for prognosis.^[18,26,28]^

Unden et al showed that an increase in serum S100B was higher in BM than in viral meningitis. Serum S100B levels were generally higher in patients with CNS infections than in those without CNS infections.^[17]^ Spinella et al reported that CSF S100B levels were elevated in children with meningitis compared to controls.^[30]^ Another work showed that in adult patients with meningoencephalitis, CSF S100B levels can be used to determine the severity of the clinical condition.^[26]^ In the study by Rohlwink et al, CSF S100B levels were high in children with TBM and were associated with death.^[31]^ Our study found high AUC values between the groups in the ROC analysis of CSF S100B levels, which can differentiate infectious meningitis groups from each other. The parameter that had the greatest additive to the discrimination of the meningitis groups from the control group was S100B, and S100B levels were significantly higher in the TBM group than in the other groups. In both the diagnosis of infectious meningitis groups and in the separation of meningitis species, the VIP score was determined to be the highest S100B. This was seen in the results showing the contribution ratings of parameters with different importance analysis in the AM, TBM, and BM groups.

Jesse et al^[32]^ suggest that GFAP has high potential as an additional diagnostic marker between BM and viral meningitis. In another study, GFAP levels in the CSF of children with TBM were high and had an association with death.^[31]^ In our study, DEFA1 and GFAP levels were higher in the patient groups than in the control groups. However, our results did not differentiate between the meningitis groups. In addition, CSF DEFA1 and GFAP levels in the heat map were also successful in discriminating between the control and the meningitis groups.

Consistent with these data, ALOX5, S100B, DEFA1, and GFAP were partially increased in the meningitis groups versus the healthy control. Here, ALOX5 and S100B were significantly increased in the TBM group compared to the other groups in the heat map. This distinction suggests that S100B may be an important biomarker in suspected cases of meningitis and in the diagnostic differentiation of TBM from other infectious meningitis cases. It may be used in addition to routine serum and CSF findings. It is clear that some new biomarkers will be useful in the rapid differential diagnosis of suspected meningitis in addition to routine CSF tests (protein, glucose, and white blood cell count) to reduce mortality and morbidity from infectious meningitis.

### 4.1. Limitations

The number of patients in our tuberculosis and aseptic meningitis groups was small. There was only one death among our patients, and it was not possible to evaluate the relationship between biomarkers and mortality across all types of meningitis. Biomarkers were measured only at disease onset, not at different disease stages. CSF levels of these markers were not compared with serum levels or other inflammatory markers. In addition, these markers were not analyzed on the basis of radiological images to assess the severity of the disease. Factors causing an increase in bacterial meningitis, CSF Gram stain and acid-fast stain results were not evaluated.

## 5. Conclusions

This study showed that the parameters ALOX5, S100B, DEFA1, and GFAP could discriminate between meningitis patients and healthy controls, although with varying degrees of reliability. The S100B protein was found to be effective in discriminating between healthy controls and patients with meningitis and between different types of meningitis. S100B plays a particularly an important role in distinguishing TBM from other types of meningitis. This protein was the most effective in separating the meningitis groups from the control group. ALOX5 can effectively separate BM from AM. These results can be used together with routine CSF parameters in clinical examinations where there is uncertainty in the rapid differential diagnosis of meningitis and meningitis types. However, large-scale studies with more cases and the exclusion of other confounding factors are needed.

## Acknowledgments

For her contribution in collecting some of the patients’ data, we would like to thanks Dr Emine Ayça Akdemir and Dr İrfan Binici.

## Author contributions

**Data curation:** Ali İrfan Baran, Şükrü Akmeşe, Hasan Karsen, Zekiye Hakseven Karaduman, Tayyar Tarcan.

**Formal analysis:** Ali İrfan Baran, Şükrü Akmeşe, Hasan Karsen, Zekiye Hakseven Karaduman, Tayyar Tarcan.

**Investigation:** Ali İrfan Baran, Şükrü Akmeşe.

**Methodology:** Ali İrfan Baran, Hasan Karsen.

**Resources:** Ali İrfan Baran, Hamit Hakan Alp.

**Visualization:** Şükrü Akmeşe.

**Writing – original draft:** Ali İrfan Baran, Zübeyir Huyut, Mehmet Reşit Öncü, Halil İbrahim Akbay, Hamit Hakan Alp.

**Writing – review & editing:** Ali İrfan Baran, Zübeyir Huyut, Mehmet Reşit Öncü, Halil İbrahim Akbay, Hamit Hakan Alp.
